# An effective combination of codon optimization, gene dosage, and process optimization for high-level production of fibrinolytic enzyme in *Komagataella phaffii (Pichia pastoris)*

**DOI:** 10.1186/s12896-020-00654-7

**Published:** 2020-12-04

**Authors:** Zhiqun Che, Xiaoyan Cao, Guiguang Chen, Zhiqun Liang

**Affiliations:** 1 State Key Laboratory for Conservation and Utilization of Subtropical Agro-bioresources, Guangxi Microorganism and Enyme Research Center of Engineering Technology, College of Life Science and Technology, Guangxi University, Nanning, 530004 China; 2 College of Agriculture and Forestry Science, Linyi University, Linyi, 276000 China

**Keywords:** Fibrinolytic enzyme, Recombinant engineered strain, Real-time quantification PCR, Fermentation optimization, Characteristics

## Abstract

**Background:**

As a main drug for diseased thrombus, some clinically used thrombolytic agents have various disadvantages, safer novel thrombolytic agents are of great demand. This study aimed to achieve high and efficient production of a fibrinolytic enzyme with superior enzymatic properties, by a combination strategy of codon optimization, gene dosage and process optimization in *Komagataella phaffii (K. phaffii).*

**Results:**

After codon optimization, the fibase from a marine *Bacillus subtilis* was expressed and secreted in *K. phaffii* GS115. Recombinant strains harboring different copies of the *fib* gene (*fib*-nc) were successfully obtained via Geneticin (0.25–4 mg/ml) screening on minimal dextrose selection plates and assessment via real-time quantitative PCR. The respective levels of fibase produced by strains expressing *fib*-5.4c, *fib*-6c, *fib*-8c, *fib*-9c, and *fib*-12c were 4428, 5781, 7323, 7930, and 2472 U/ml. Levels increased as the copy number increased from 4 to 9, but decreased dramatically at copy number 12. After high cell density fermentation optimization, the highest fibase activity of the strain expressing *fib*-9c was 7930 U/ml in a shake flask and increased to 12,690 U/ml after 3 days of continuous culture in a 5-L fermenter, which is one of the highest levels of production reported. The recombinant fibase was maximally active at pH 9.0 and 45 °C, and was remarkably stable at pH levels ranging from 5 to 10 and temperatures up to 50 °C. As a metal-dependent serine protease, fibase did not cause hemolysis in vitro and preferentially degraded fibrin directly.

**Conclusions:**

The combination of codon optimization, gene dosage, and process optimization described herein could be used for the expression of other therapeutic proteins difficult to express. The characteristics of the recombinant fibase suggest that it has potential applications for thrombosis prevention and therapy.

## Background

In recent years increasing numbers of cardiovascular diseases caused by thrombosis have begun to contribute to impairment of human health, and they are the main reason for morbidity and mortality worldwide [1]. As the main medical drug for diseased thrombus, thrombolytic agents such as urokinase, streptokinase, and tissue-type plasminogen activator have been widely used in clinical therapy. There have always been associated disadvantages however, including high cost, short half-lives, low fibrin specificity, bleeding complications, and allergic responses [2]. There is therefore a demand for safer novel thrombolytic agents. Many fibrinolytic enzymes (fibases) from animals such as snakes [3], earthworms [4, 5], plants [6], the fungus *Cordyceps militaris* [7] and microbes [8, 9] have been discovered and studied extensively. However, there are few reports on fibase produced by marine microorganisms.

Microbial fibases are of great interest to researchers due to their potential for use in thrombolytic therapy, which would be of high yield, low cost, and amenable to industrial scale-up. Over-expression of foreign genes in *Escherichia coli* (*E. coli*) commonly leads to the formation of inclusion bodies, resulting in inactive proteins. As an expression system, *Bacillus subtilis* (*B. subtilis*) has significant advantages over *E. coli* in that proteins can be readily secreted [10]. But *B. subtilis* also exhibits high protease expression leading to degradation of target proteins [11], and this high protease expression increases the downstream purification difficulty of target protein. To date two main aspects of protein expression in wild *B. subtilis* have been investigated. One is the generation of a *B. subtilis* strain rendered deficient in protease expression via gene knock-out [12, 13]. The other is optimization of fermentation processes with respect to high protein expression. The use of yeasts is conducive to facile genetic manipulation and ease of fermentation of the microorganism, which can result in high yields of proteins in a solution that does not contain pyrogens, pathogens, or viral inclusions [14]. Their rapid growth, microbiological safety, and amenability to high-density fermentation in simple media render them particularly suited to large-scale industrial production of foreign proteins, where secretory expression is important for simplifying the downstream protein purification process [15]. In recent years, as one of the most important industrial organisms for heterologous protein production, *Komagataella phaffii (K. phaffii)* has been used widely for the production of a broad range of recombinant drug products, including antibody fragments [16], growth factors [17], interferon [18], interleukin [19], insulin precursors [20], and staphylococcal kinase [21] etc.

Despite its high protein productivity, further optimization of *K. phaffii* expression is imperative due to strain-specific and product-specific challenges such as promoter strength, methanol utilization type, and oxygen demand. To this end, strategies including genetic and process engineering have been employed in this study. Optimizations of codon use and gene dosage have been proved useful for enhancing protein expression levels. Codon optimization includes the adjustment of codon usage bias and GC content, repeat sequence removal, and undesired sequence motif modification. All these factors could render its mRNA secondary structures more stable, increase the translation efficiency and prolong the half-life of mRNA, and finally result in an increase of protein synthesis [22]. Gene dosage (i.e., the RNA abundance at the transcriptional level) is a major limiting factor for high and efficient expression of the targeted gene. Increasing the gene dosage usually has substantial effects on the improvement of protein expression levels [23, 24], and multiple copies of the foreign gene can be inserted at the same site in yeast genomes. Recombinant yeast strains with high expression profiles can be generated by optimizing the number of foreign gene copies inserted. Large-scale production of proteins via high cell density fermentation relies on the optimization of process parameters including methanol feed rate, induction temperature, and specific growth rate [22].

Currently recombinant *K. phaffii* strains with high copy numbers can only be obtained by increasing gene concatemers, so alternative methods for generating multicopy strains rapidly and reliably are highly desirable [25]. Additionally, in order to study the effects of different promoters on protein expression levels, it is necessary to accurately and quantitatively analyze the copy number of foreign genes inserted into the recombinant yeast. In the process of strain propagation, reduced copy number can indicate plasmid loss. Real-time quantification PCR (qPCR) has been used to detect the copy numbers of many foreign genes including transglutaminase [25], human interleukin-3 [26], and lipase [27] in recombinant yeasts, but to date it has not been used in *fib* gene copy number estimation.

In the current study, after codon optimization, the fibase from a marine *B. subtilis* D21 was expressed and secreted in *K. phaffii* GS115. Multicopy integrants were obtained via screening and qPCR method to enhance the expression of fibase. Shake flask production series were conducted to optimize cultivation parameters such as medium composition, incubation time, temperature, methanol concentration, and coculture with methanol and sorbitol. After high cell density fermentation optimization, the highest fibase activity of the strain expressing *fib*-9c was 7930 U/ml in a shake flask and increased to 12,690 U/ml after 3 days of continuous culture in a 5-L fermenter, which is one of the highest levels of production reported. Then fibase was purified using one-step Ni-NTA and its biological characteristics was evaluated.

## Methods

### Strains

The strains and plasmids used are shown in Table 1. *E. coli* was cultivated in Luria-Bertani medium at 37 °C, and *K. phaffii* was cultured in buffered glycerol-complex medium (BMGY, 1% yeast extract, 2% peptone, 100 mM potassium phosphate buffer, 4 × 10^− 5^% biotin, 1.34% Yeast Nitrogen Base, 1% glycerol, pH 6.0) for growth, buffered methanol-complex medium (BMMY, 1% yeast extract, 2% peptone, 100 mM potassium phosphate buffer, 4 × 10^− 5^% biotin, 1.34% Yeast Nitrogen Base, 0.5% methanol, pH 6.0) for fibase induction, or yeast extract peptone dextrose (YPD) free of glucose (1% yeast extract and 2% peptone) for growth and induction.

**Table 1:** The strains and plasmids used in the current study

	Characteristics	Source
Strains		
*B. subtilis* D21	Wild-type strain with fibrinolytic activity	Lab stock
*K. phaffii* GS115	His4, host strain, methylotrophic	Lab stock
Plasmids		
Topo-*fib*	Topo harboring an internal 825-bp *fib* gene fragment	Current study
pMD19T-*TDH1*	pMD19T harboring an internal 1002-bp *fib* gene fragment	Current study
pPIC9K	*E. coli* and *K. phaffii* shuttle vector; Amp^r^, G418^r^ containing AOX1 promoter for tightly regulated, methanol-induced expression of the gene	Invitrogen
pPIC9K-*fib*	pPIC9K derivative harboring an internal 825-bp *fib* gene fragment	Current study

### Construction, transformation, and screening for recombinant *K. phaffii*

**Fig. 1:**
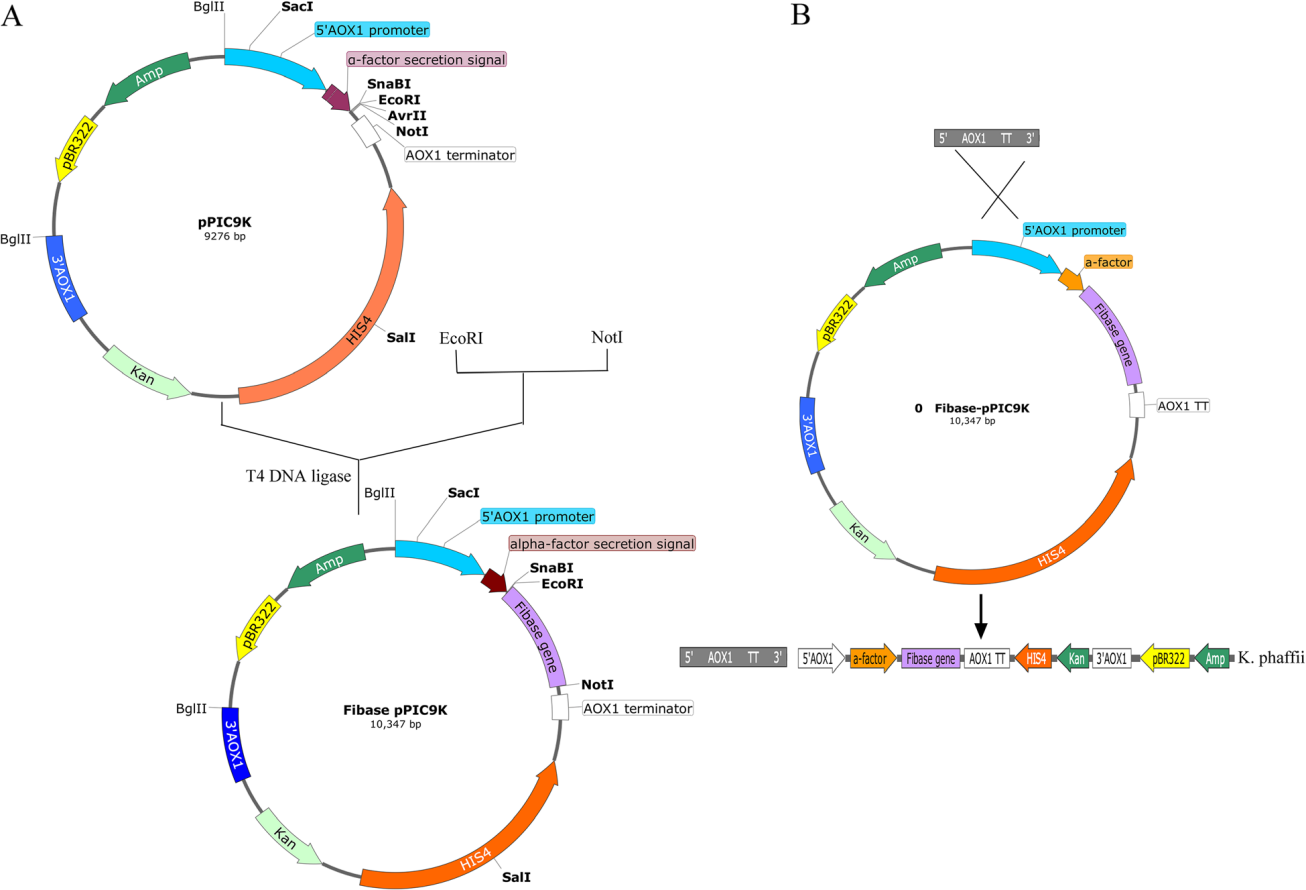
Construction of recombinant plasmid pPIC9K- *fib* and its integration sites in the genome

Th*e fib* gene (GenBank: KM519994.1) used in this study was derived from a marine *B. subtilis* D21 screened previously. For high production, 168 codons in total (approximately 61.1%) were replaced with the *K. phaffii*-preferred codons (Fig. S1). The codon adaptation index was increased from 0.64 of initial sequence to 0.96 of optimized sequence (a codon adaptation index of 0.8–1.0 is regarded as good for high expression). The optimized *fib* fragment was digested by *Eco*RI/*not*I and then cloned into the simultaneously digested pPIC9K vector, together with the a-factor signal peptide under control of the AOX1 promoter. The recombinant plasmid pPIC9K-*fib* was introduced into competent *E. coli* DH5α by chemical transformation, positive clones were selected by ampicillin (Amp, 100 μg/ml) resistance, colony PCR and sequencing. The recombinant vector pPIC9K-*fib* was linearized with *Sac*I and transformed into competent *K. phaffii* GS115 cells by *electroporation,* using the set program with a *voltage* of 1500 V, 25 μF *capacitance*, and 200 Ω *resistance*. The construction of recombinant pPIC9K-*fib* is depicted in Fig. 1a, and the process of integration into the genome is depicted in Fig. 1b. The single *copy or high copy recombinant K. phaffii GS115/pPIK9K-fib* were selected using minimal dextrose selection plates containing *different concentrations of* Geneticin (G418, 0.25 mg/ml of 1 copy, 0.5 mg/ml of 1–2 copy, 4 mg/ml of 7–12 copy), colony PCR and sequencing, which were inoculated into glucose-free YPD or BMGY at 30 °C 200 r/min for 24 h, then transferred to BMMY for induce expression.

### Analysis of *fib* copy number by real-time quantification PCR

Five positive clones with different fibase activities were selected for analysis of *fib* copy numbers, which were estimated via qPCR in the *K. phaffii* genome. The primers and probes used for plasmid construction and qPCR are as shown in Table 2. There is only a single copy of the housekeeper gene *TDH1* in the *K. phaffii* genome [28], so the *TDH1* copy number can be used to represent the initial copy number of the genome in the template. After the recombinant plasmid Topo-*fib* and pMD19T-*TDH1* were respectively linearized with *Xba*I and *Bam*HI as standards, the absolute copy number of the target gene and reference gene were calculated using Eq. 1 below. The standard curve was constructed using real-time fluorescence quantitative Ct values and the logarithm of the copy number of the standard (copies/μl) after gradient dilution. Using genomes with the highest fibase activity and the aforementioned other four positive clones as sample templates, the absolute copy numbers of the target and reference genes were calculated from Ct according to the standard curve, then the copy number of the target gene was calculated using Eq. 2 below.

$$ {\mathrm{Copy}\ \mathrm{number}}_{\left(\mathrm{test}\ \mathrm{sample},\kern0.75em TDH1\ \mathrm{and}\  fib,\kern0.75em \mathrm{copies}/\mathrm{ul}\kern0.5em \right)}=\frac{{\mathrm{OD}}_{260}\times 50\times \mathrm{Dilution}\ \mathrm{fold}\times 6.022\times {10}^{14}}{640\times \mathrm{Number}\ \mathrm{of}\ \mathrm{plasmid}\ \mathrm{bases}}\kern1em (1) $$

$$ {\mathrm{Copy}\ \mathrm{number}}_{\left(\mathrm{target}\  gene,\kern0.75em fib\right)}=\frac{\mathrm{Absolute}\ \mathrm{copies}\left(\mathrm{target}\ \mathrm{gene}, fib\right)}{\mathrm{Absolute}\ \mathrm{copies}\left(\mathrm{reference}\ \mathrm{gene}, TDH1\right)}\kern1em (2) $$

**Table 2:** Primers and probes used for plasmid construction and real-time quantitative PCR

	Sequence (5′ → 3′)	Annotation
*fib*-F	GAATTCGCAGGGAAATCAAACGGG	PCR for pPIC9K-*fib*
*fib*-R	GCGGCCGCTTAGTGGTGATGATGGTGATGTTGAGCAGCAGCTTGAACGT	PCR for pPIC9K-*fib*
*fib*-F	GCAGGGAAATCAAACGGG	PCR for pMD19T-*fib*
*fib*-R	TTGAGCAGCAGCTTGAACGT	PCR for pMD19T-*fib*
*TDH1*-F	ATGGCTATCACTGTCGGTATTAACG	PCR for pMD19T-*TDH1*
*TDH1*-R	TTAAGCCTTAGCAACGTGTTG	PCR for pMD19T-*TDH1*
q*fib*-F	TTGCCGGTGGAGCTTCTATG	qPCR for *fib*
q*fib*-R	CAACAGAGTTGTTCAAAGCAGCA	qPCR for *fib*
Probe-*fib*	HEX-TCCATTCCAAGATTACAACTCTCATGG-BHQ-1	qPCR for *fib*
q*TDH1*-F	CGGTGTTTTCACCACTTTGGA	qPCR for *TDH1*
q*TDH1*-R	CAACGAACATTGGAGCATCCT	qPCR for *TDH1*
Probe-*TDH1*	HEX-CCAAAAGCACATCGACGCCGGT-BHQ-1	qPCR for *TDH1*

### Fermentation optimization in flasks and 5-L fermenter

In order to establish an efficient and low-cost process for recombinant fibase production, the medium (glucose-free YPD and BMMY), time-course (1–9 days), temperature (28 °C, 32 °C, and 36 °C), methanol concentration for induction (0.5, 1, and 2.5%), and coculture of methanol and sorbitol (0, 2, 4, and 6 g/l) conditions were optimized via an extensive series of shake flask experiments. The shake flask study under optimized condition was performed in 50 ml media in 250 ml shaker flasks. Methanol was added every 24 h to different final concentration to maintain induction. The results of optimization were then verified in scaled up production in a 5-L fermenter with 2.4 l medium volume. The main parameters utilized during the entire fermentation process are shown in Table S1. All values are means ± SD from three independent experiments.

### Determination of fibrinolytic activity and protein concentration

Fibrinolytic activity was assayed via the standard fibrin plate method using urokinase [29], with some modifications. Each 10-ml plate contained 0.1% fibrinogen, 1% agarose, and 10 U thrombin. A total of 5 μl of culture supernatant at various dilutions was added to each well. The plate was incubated for 18 h at 37 °C, and fibrinolytic activity was determined based on the standard curve of urokinase. One enzyme unit is defined as the enzyme amount producing a ΔA275 of 1.0 per ml per minute at 37 °C, pH 7.5, when measuring perchloric acid soluble products from α-casein. All data are presented as the mean ± SD of triplicate determinations. The concentration of recombinant fibase was measured using nucleic acid/protein analyzer.

### Purification and sodium dodecyl sulfate polyacrylamide gel electrophoresis (SDS-PAGE)

Utilizing the 6His tag at the C-terminal end the recombinant protein was concentrated using an ultrafilter tube and purified with nickel ion affinity chromatograph resin (Ni-NTA) to obtain a single band. The buffer systems utilized were Ni-50 wash buffer (50 mM imidazole, 300 mM NaCl, 50 mM NaH_2_PO_4_, pH 8.0), Ni-250 elution buffer (250 mM imidazole, 300 mM NaCl, 50 mM NaH_2_PO_4_, pH 8.0), cleaning-in-place buffer (0.5 M NaOH), stripping buffer (50 mM Na_3_PO_4_·12H_2_O, 300 mM NaCl, 100 mM EDTA·Na_2_), and regeneration buffer (100 mM NiSO_4_·6H_2_O). All purification processes were conducted at 4 °C. Purification recovery refers to the ratio of total activity before and after purification.

SDS-PAGE was conducted on a Mini-PROTEAN 3 cell apparatus (Bio-Rad, USA) using the tris-glycine discontinuous system with 5% stacking and 12% resolving gels. An unstained protein marker, ProteinRuler II (TRAN, 12–120 kDa) was used as molecular weight standards. Proteins were stained using the Coomassie Brilliant Blue R-250 method.

### Characteristics of recombinant protein

Residual fibase activity under the different treatments was determined via the fibrin plate method. The determination of optimal temperature was performed by measuring fibase activity in 0.02 M phosphate-buffered saline (pH 7.4) from 28 °C to 60 °C. The thermal stability of fibase was determined after incubation in the same buffer and temperatures for 1 h or 6 h.

To determine the optimum pH, enzymatic activity measurements were performed at levels ranging from pH 4.0 to pH 10.0. To investigate the pH stability of the purified enzyme, enzyme samples were diluted to the same concentration with 0.02 M disodium hydrogen phosphate-sodium citrate (pH 4.0–6.0), 0.02 M Tris-HCl (pH 7.0–8.0), and 0.02 M glycine-sodium hydroxide (pH 9.0–10.0). Residual fibase activity was measured after incubation at 37 °C for 18 h.

Salt solutions containing different metal ions were prepared, and purified enzymes were added to 5, 20, and 50 mM salt solutions. The residual fibase activity of each enzyme was determined via fibrin plates. Fibase activity with no salt was set as 100%.

The kinetic parameters K_m_ and V_max_ for fibase were calculated using chromogenix as the substrate at concentrations of 0.0, 0.1, 0.3, 0.6, 0.9, and 1.2 mM. The kinetic study was performed at 37 °C and pH 7.4 (phosphate buffer, 0.02 M). Fibase was incubated with substrates at different concentrations at 37 °C for 8 min, and absorption values at 405 nm were measured. The amount (μM) of 4-nitroanilide released by the reaction between tetra-peptide substrate and protease per unit of time was considered to be the velocity of the reaction.

All values above are mean ± SD from three independent experiments.

## Results

### Expression of yeast codon optimized strains

*K. phaffii* is a methylotrophic yeast without fibrinolytic activity, the expression efficiency of which is higher than that of *Saccharomyces cerevisiae* due to the tight regulation and strong inducibility of the AOX1 promoter when methanol is used as the sole carbon source [22]. The recombinant strain GS115 (pPIC9K-*fib*) was cultivated with a 2% inoculum size and daily addition of 1% (v/v) methanol to a shaking flask containing 50 ml BMMY at pH 6.0 and 30 °C*.*

**Fig. 2:**
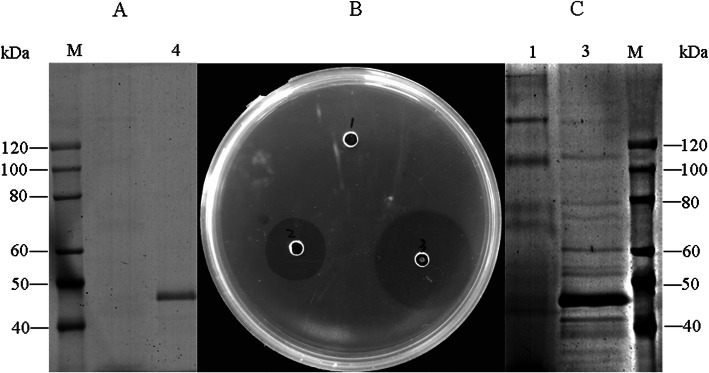
Effects of codon optimization on fibase activity and yield. After 5 days of cultivation the culture supernatant was harvested and analyzed via a fibase activity assay (**b**) and SDS-PAGE (**c**). The fibase purified via one-step Ni-NTA is 46 kD (**a**). 1: GS115 (pPIC9K). 2: 125 U/ml urokinase. 3: GS115 (pPIC9K-*fib*). 4: Purified fibase. M: Protein Ruler II

A main protein band with a molecular weight of 46 kDa was detected via SDS-PAGE of positive culture supernatants with the highest fibase activity after supernatant concentration (Fig. 2c). No corresponding band was yielded by the transformant with pPIC9k, and the result was consistent with that of fibrin plates (Fig. 2b). Therefore, recombinant fibase was secreted into the supernatant by *K. phaffii* GS115. The 6His tag at the carbon end facilitated purification of the recombinant protein via nickel ion affinity chromatograph resin, and it was analyzed via SDS-PAGE (Fig. 2a).

### Quantification of *fib* copy number in *K. phaffii* genome

**Fig. 3:**
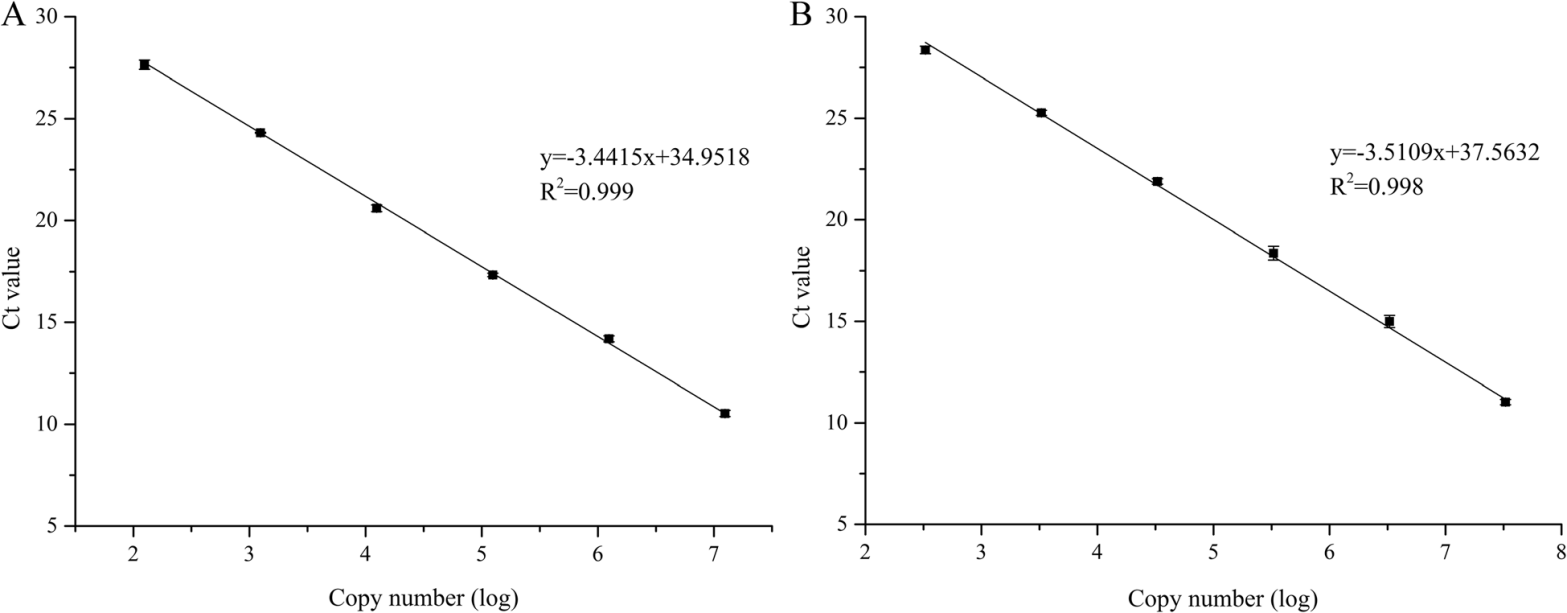
Standard curves of the reference gene *TDH1* (**a**) and the target gene *fib* (**b**). Data are mean ± SD of three independent runs

The melt curves of both *TDH1* and *fib* only had a single peak, indicating specificity of the qPCR products (Fig. S2A and S2B). The amplification efficiency of *TDH1* was 94.8%, and that of *fib* was 96.3%. The regression equation of the *TDH1* standard curve was y = − 3.441x + 34.952 (R^2^ = 0.999), and that of *fib* was y = − 3.511 x + 37.563 (R^2^ = 0.998) (Fig. 3). The *fib* copy numbers in the recombinant strain with the highest activity and the other four strains were 5.4, 6.0, 8.0, 8.7 and 12.3 (Table 3). The corresponding strain were named GS115/*fib*-5.4c, *fib*-6c, *fib*-8c, *fib*-9c, and *fib*-12c with fibrinolytic activity 4428 U/ml, 5781 U/ml, 7323 U/ml, 7930 U/ml, 2472 U/ml respectively.

**Table 3:** Fibrinolytic enzyme activity and *TDH1* and *fib* gene copy numbers detected via real-time quantitative PCR

Strain	Fibase activity	Ct value		Gene copy (10^*n*^)		Copy numbers of *fib* gene (*fib*/*TDH1* copy number)
		*fib* gene	*TDH1* gene	*fib* gene	*TDH1* gene	
GS115/ *fib*-5.4c	4428	26.00 ± 0.12	26.22 ± 0.59	18.6 ± 1.46	3.45 ± 1.20	5.4
GS115/ *fib*-6c	5781	16.13 ± 0.02	16.99 ± 0.23	14.9 ± 1.88	2.50 ± 0.42	6.0
GS115/ *fib*-8c	7323	16.00 ± 0.19	16.40 ± 0.24	13.4 ± 0.14	1.68 ± 0.27	8.0
GS115/ *fib*-9c	7930	16.23 ± 0.04	17.19 ± 0.07	12.6 ± 0.32	1.45 ± 0.07	8.7
GS115/ *fib*-12c	2472	26.35 ± 0.19	27.71 ± 0.19	14.8 ± 1.88	1.21 ± 0.15	12.3

Data are presented as the mean ± SD of triplicate determinations

### Fermentation optimization in flasks and 5-L fermenter

**Fig. 4:**
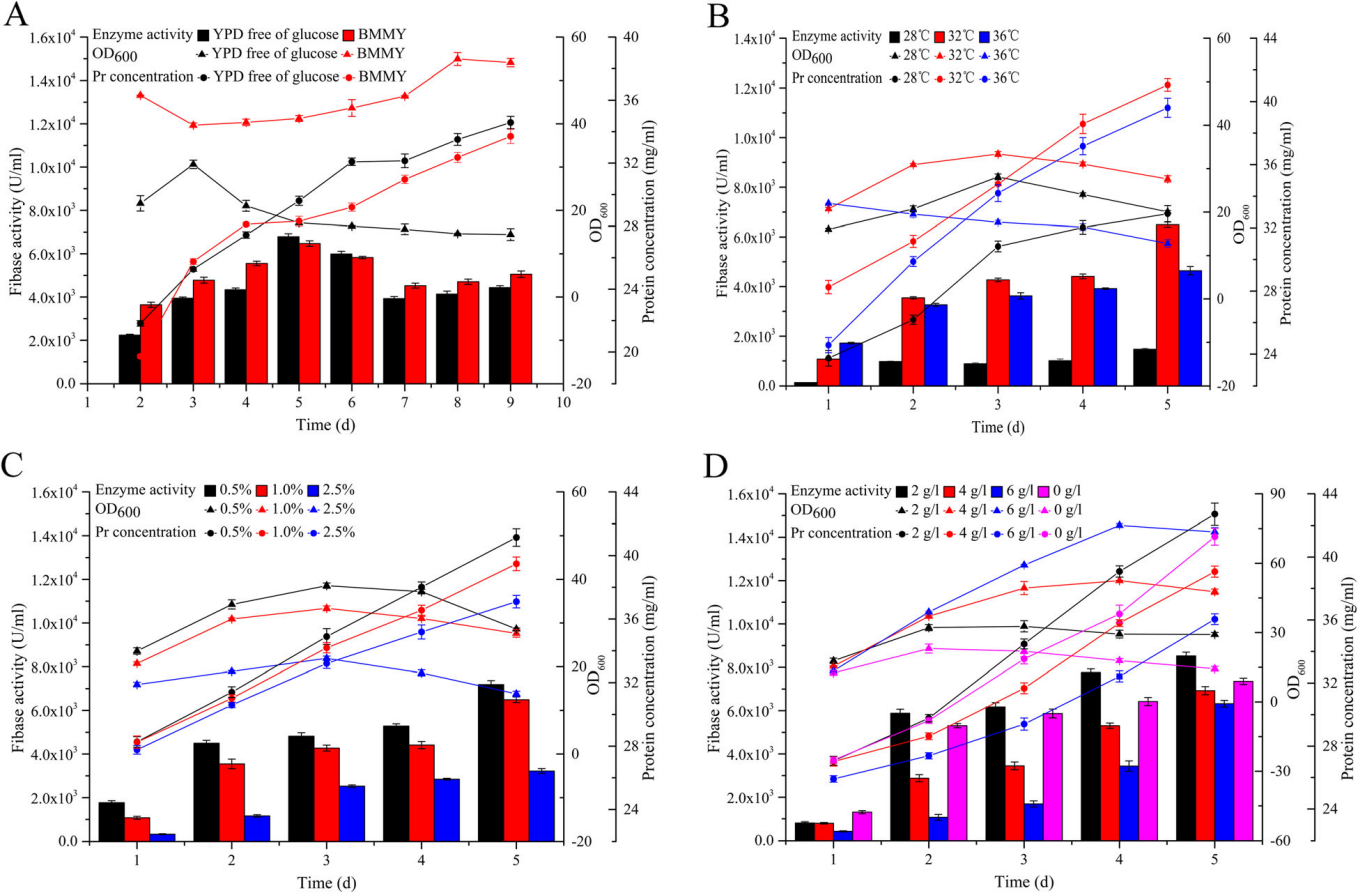
Cultivation optimization of recombinant strain GS115/pPIC9K-*fib* in shaking flasks. The figures show the effect of different media and days (**a**), induction temperatures (**b**), methanol concentrations (**c**), coculture with methanol and sorbitol (**d**) on enzyme activity (column), growth (line and triangle), and protein concentration (line and circular), respectively. All values are mean ± SD from three independent experiments

**Fig. 5:**
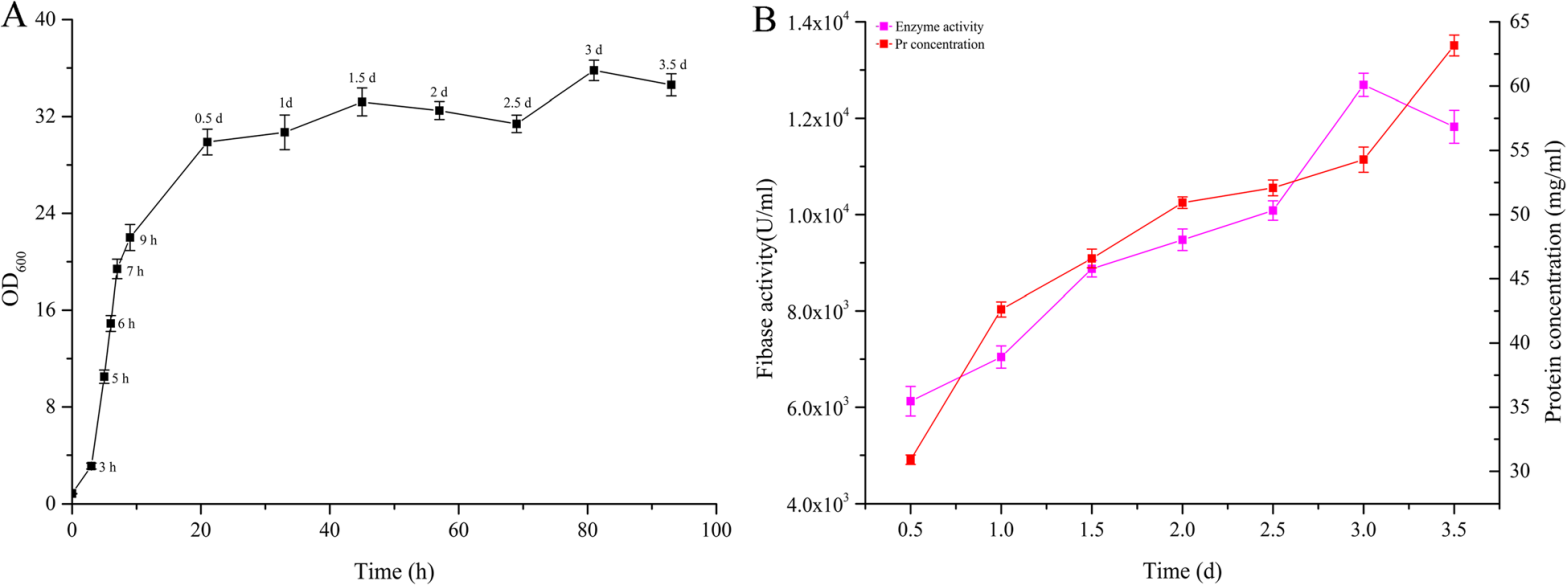
The growth, enzyme activity, and protein concentration from fermentations of recombinant strain GS115/pPIC9K-*fib* in 5-L fermenter. Data are presented as mean ± SD of triplicate experiments

After optimization the maximum fibase activity was obtained with a 2% inoculum size, and the optimal culture conditions included daily addition of 0.5% (v/v) methanol and 2 g/l sorbitol to a 250 ml shaking flask containing 50 ml of glucose-free YPD (1% yeast extract and 2% peptone) culture medium for 120 h at pH 6.0 and 32 °C (Fig. 4). Based on the optimized conditions of fibase recombinant strain cultivation in shaking flasks, the fermentation process in the 5 L-fermenter was established. The highest fibase expression level of the strain expressing *fib*-9c was 7930 U/ml in a shake flask and increased to 12,690 U/ml after 3 days of continuous culture in a 5-L fermenter (Fig. 5).

### Characteristics of recombinant protein

Effects of pH and temperature on fibase activity and stability.

**Fig. 6:**
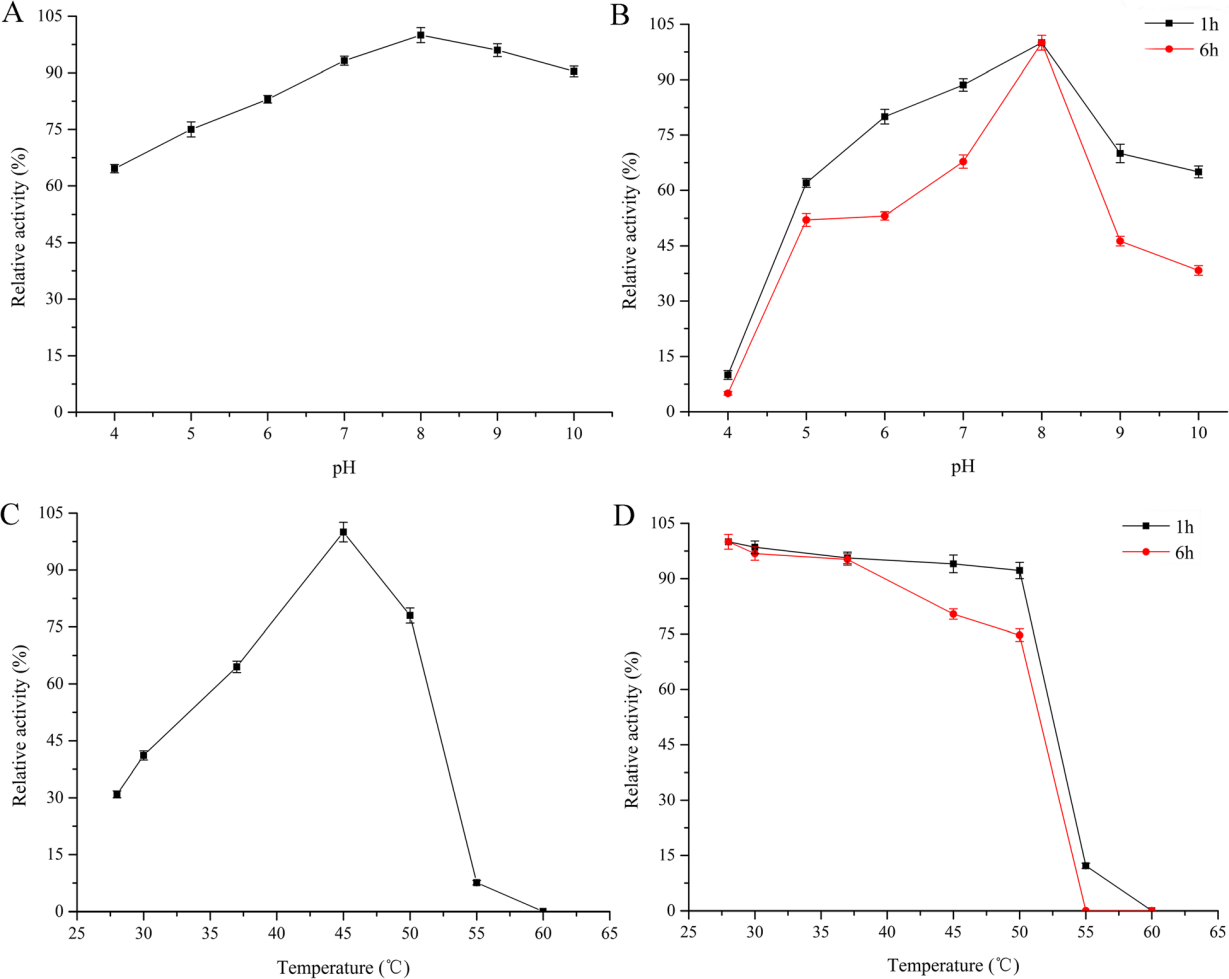
Effects of pH and temperature on fibrinolytic enzyme activity and stability. All values are mean ± SD from three independent experiments

As shown in Fig. 6, the purified fibase had high relative activity (> 65% of maximum) at pH values ranging from 4.0 to 10.0 and the optimum pH was 8.0. The purified fibase exhibited maximal activity at 45 °C, and it was stable and retained over 90% of its initial activity after incubation for 1 h at temperatures between 28 °C and 50 °C. Even after incubation for 6 h at 50 °C the residual relative activity was > 75%.

### Effects of metal ions on fibase activity

The effects of different metal ions on fibase activity as determined via fibrin plates after dilution to various different concentrations are shown in Table 4. With the exception of activation by Ca^2+^ and Mg^2+^, all other metal ions investigated inhibited fibase activity.

**Table 4:** Effects of metal ions on fibrinolytic enzyme activity

Metal ions	Concentration relative activity (%)^a^		
	5 mM	20 mM	50 mM
Ca^2+^	117.5 ± 1.8	121.2 ± 2	97.1 ± 1.2
Mg^2+^	100.5 ± 1	108.9 ± 1.4	98.0 ± 2.1
Zn^2+^	8.1 ± 1.4	4.5 ± 1.2	1.8 ± 1.0
Cu^2+^	5.4 ± 1.9	0.0 ± 1.0	0
Mn^2+^	88.3 ± 1.6	7.3 ± 1.4	0
Pb^2+^	0	0	0
Fe^3+^	0	0	0
CK^b^	100	100	100

^a^The relative activity with different metal ions added was determined and compared with the activity measured in phosphate buffer (pH 7.4, 0.02 M) without the addition of any ions

^b^The relative activity measured in phosphate buffer (pH 7.4, 0.02 M) without the addition of any ions

All values are mean ± SD from three independent experiments

### Kinetic studies

The kinetic parameters K_m_ and V_max_ for fibase calculated using chromogenix substrate s-2251 as a substrate were 2.7 mmol/l and 0.03 mmol/l^/^min respectively (Fig. S3). The lower K_m_ value of the purified fibase in the present study indicated that it had a greater affinity for the substrate.

### Mechanisms of fibrinolytic effects and in vitro hemolysis assays

**Fig. 7:**
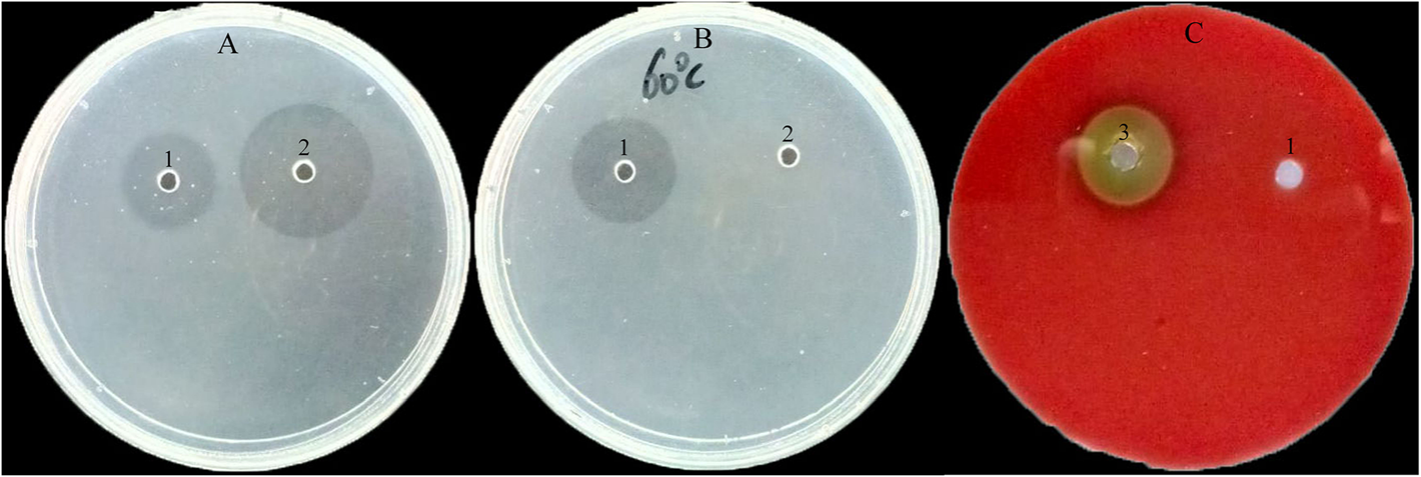
Mechanisms of fibrinolytic effects (**a**, **b**), and the hemolysis assay in vitro (**c**). In contrast to the urokinase (2), the purified enzyme (1) generated translucent zones of equal size on fibrin plates containing both plasminogen and fibrinogen (**a**) and fibrinogen only (**b**). The purified enzyme did not generate a translucent zone on a blood agar thus it did not induce hemolysis in vitro, unlike the crude extract (3)

As shown in Fig. 7a and b the sizes of the translucent zones generated by purified enzyme on the two plates were very similar, but urokinase did not generate a translucent zone on the heated fibrin plate, suggesting that the mechanism of action of purified enzyme involves direct thrombolysis. Purified fibase did not form a translucent zone on the bovine blood agar plate, unlike the crude enzyme, indicating that the purified fibase did not induce hemolysis in vitro (Fig. 7c). Therefore, it may be a potential thrombolytic agent with low bleeding risk for safe therapy.

## Discussion

Codon optimization is a key efficient strategy and measure to improve the expression, which has been repeatedly demonstrated to increase heterologous protein expression in *K. phaffii*. The expressions of a codon-optimized *lip2* gene from *spergillus niger* was enhanced 11.6-fold compared to the native gene [30]. Codon optimization of the endoinulinase gene resulted in 4.8-fold increases in enzyme activity [31]. Compared with the expression level of native hBMP4 sequence (12 mg/L), there is a 4-fold increase in that from the optimized sequence (48 mg/L) [32]. There are several factors remarkably affecting the expression of foreign genes in *K. phaffii*, containing codon usage bias, GC content, transcriptional and translational blocks, and secondary structure of mRNA et al. The optimization of these factors could finally result in an increase of protein synthesis.

The *fib* gene used in the current study was originated from a marine *B. subtilis* isolated from a coastal mangrove area in Beihai City in Guangxi, China. Marine microorganisms can survive in the extreme ocean environment, wherein the pH is similar to that of the internal environment of the human body. Therefore, marine microbes are considered a rich source of bioactive compounds that have high medicinal value [33, 34]. Since the 1960s thousands of novel secondary metabolites with antitumor, antiviral, antibacterial, antifungal, and anticoagulant activities have been isolated from marine microorganisms [35]. The recombinant fibase remained active over a broad pH (6.0–10.0) with optimum pH 8.0, which is very close to the physiological pH. Its optimum pH is quite different from many other fibrinolytic enzymes, but similar to fibrinolytic enzymes derived from high-salt environments, such as seas [36] and douchi [37]. Additionally, the purified fibase had high tolerance to the increasing temperature. As a metal-dependent serine protease, fibase did not cause hemolysis in vitro and preferentially degraded fibrin directly. All these characteristics exhibited its desirable adaptation to the internal environment of human body and great potential for application in thrombosis prevention and/or therapy.

An additional yield-limiting factor of recombinant protein is the efficiency of purification and recovery. Conventionally, separation and purification of wild fibase relies on methods like phenylene anion exchange, gel filtration, and hydrophobic interaction chromatography et al., which are complex operations resulting in low yields. Here, the fibase was purified by one-step Ni-NTA, achieving a 27 ± 2% recovery. The recovery was much higher than those of other fibrinolytic enzymes ever reported, such as 7.5% recovery from *B. subtilis* ICTF-1 [38], 6.46% recovery from Chive (*Allium tuberosum*) [6], and 7.82% recovery from *Pseudomonas baetica* SUHU25. This method is highly efficient and suited for large-scale purification of target protein.

Heterologous gene copy number reportedly plays an important role in the yield of recombinant proteins in *K. phaffii* expression systems [39]. In recent studies the activity of recombinant protein increased as the copy number increased from 4 to 9, but it decreased dramatically when the copy number continued to increase. That is a result of the negative effects of a high concentration of target protein on the growth and metabolism of the strain [40–42]. Song et al. (2019) reported that a co-expressing strain (pro/rDNA-*mtg*) with three copies of *mtg* genes (*mtg*-3c) exhibited higher transglutaminase activity than *mtg*-2c, *mtg*-6c, or *mtg*-8c [25]. Dagar et al. (2018) reported that the expression of human interleukin-3 protein increased with the addition of up to 8 copies of the expression cassettes, then drastically decreased thereafter [26]. Fang et al. (2014) reported that the respective maximum lipase activities of three recombinant strains with copy numbers of 7, 5, and 3 were 6600 U/ml, 6000 U/ml, and 4800 U/ml [27]. Therefore, the optimal copy number for high expression by *K. phaffii* needs to be evaluated via screening experiments, and it is not simply a case of “the more copies the better” [42]. After the optimal gene copy number has been evaluated, the main limitations of heterologous protein secretory expression pertain to protein translation, post-translational modification, and targeted delivery of protein precursors, which may be the main bottlenecks affecting yields [43, 44].

In order to provide a reference for the large-scale industrial production, the fermentation scale needs to be verified. There are many factors that affect fibrinolytic activity; thus the fermentation process needs prior optimization, which were initially performed at shake flask level to reduce cost and improve efficiency followed by fermenter study. As a complex and nutrient-rich medium, YPD free of glucose is prone to foaming*.* However, excessive antifoam may be toxic to cell growth, and to avoid this problem, an increase in inoculum size (10%) is required to allow cells to enter the log phase after 3 h. The rapid proliferation of *K. phaffii* resulted in faster consumption of available nutrients, and the decrease of medium foaming ability. Notably, *K. phaffii* showed transient secondary growth at 3 days, the reason of which may be that at least one nutrient had been used up and other nutrients started to be consumed. In addition, the initial induction biomass has been reported an important impact on recombinant protein yields [45]. If the cell density is too high, cell growth and metabolism will be restricted, resulting in a low enzyme activity; while if the cell density is too low, the induction phase will be much longer. The fibase activity of the strain expressing *fib*-9c was 12,690 U/ml after 3 days in a 5-L fermenter, which was 1.6-fold the highest fibase activity in shake flasks. *The clear difference in fibase yield* might attribute to the great *difference* between the fermentation conditions of fermenter and shaking flasks.

With the development of microbial technology, efficient protein expression could be achieved via genetic or engineering technologies. Some recently reported studies have investigated the homologous and heterologous expression of fibase, but their expression levels are typically low (Table 5). A study by Lv et al. overexpressed the recombinant plasmid pET-DsbA/PPFE-I in *E. coli* BL21 (DE3) and achieved maximum fibase activity of 228 U/mL after IPTG induction at 30 °C for 1.5 h, a level almost 2 times higher than that of wild strain *P. polymyxa* EJS-3 [8]. *Fib* gene (subtilisin DFE) fused with α-amylase promoter and signal peptide was successfully expressed in *B. subtilis* WB600, achieving a fibrinolytic activity of 200 U/mL. And it was 4-fold higher than that under the control of subtilisin DFE promoter [46]. Yao et al. transformed the fibase recombinant plasmid pHY300PLK/aprEBS15 into *B. subtilis* WB600 and achieved recombinant protein maximum fibrinolytic activity of 408.2 U/ml after induction at 37 °C for 96 h [47]. Several possible reasons might account for such a low production. First, the yield of recombinant fibase depends greatly on vector-host system. More specifically, high expression is generally associated with high-copy plasmids, and suitable host with high promoter activity plus high secretion efficiency. Second, the recombinant fibase might misfold into non-functional conformation, leading to aggregation or proteolytic digestion of misfolded protein. Third, the recombinant fibase may be toxic to the hosts, causing the host’s slow growth, impaired metabolism, or even death during expression. In order to improve the production of fibase in a high-efficiency *K. phaffii* expression system in the present study, various genetic and process engineering cultivation strategies were utilized at different levels. This resulted in improved fibase activity levels of up to 7930 U/ml in shake flasks and 12,960 U/ml in 5-L fermenter, which is among the highest levels of fibase production ever reported.

**Table 5:** Summary of recent reports of fibrinolytic enzyme expression in the literature

Wild-type strain	Host (strains/plasmids)	Fibase activity	References
*Paenibacillus polymyxa* EJS-3	*E. coli* BL21 (DE3)/pET-DsbA	228.0 U/ml	[8] (Lv et al. 2015)
*Bacillus amyloliquefaciens*	*B. subtilis* WB600/pSUGV4	200.0 U/ml	[46] (Xiao et al. 2004)
*Bacillus pumilus* BS15	*B. subtilis* WB600 */*pHY300PLK	408.2 U/ml	[47] (Yao et al. 2018)
*Bacillus pumilus* BS15	none	242.5 U/ml	[47] (Yao et al. 2018)
*Xanthomonas oryzae* IND3	none	2294 ± 12.8 U/g	[48] (Vijayaraghavan et al. 2019)
*Serratia* KG-2-1	none	250.4 U/ml	[9] (Taneja et al. 2017)
*Serratia rubidaea*	none	394.9 U/ml	[49] (Anusree et al. 2020)
*Bacillus sp.* IND12	none	4143 U/g	[50] (Vijayaraghavan et al. 2017)
*Cordyceps militaris*	none	120.0 U/ml	[51] (Liu et al. 2017)
*B. subtilis* D21	none	279 U/ml	This research
*B. subtilis* D21	*K. phaffii* GS115 */*pPIC9K	7930/12690 U/ml	This research

## Conclusions

To conclude, in this work, the optimized *fib* gene was expressed at different copy numbers in *K. phaffii* to study the association between fibase production and gene dosage. Relative gene copy numbers were assessed by qPCR. The results showed that, the fibase levels increased as the copy number increased from 4 to 9, but decreased dramatically at copy number 12. The fermentation optimization of the strain expressing *fib*-9c was initially done at shake flask.

level and then fermenter verification, with the maximum fibase activity 7930 and 12,690 U/ml, respectively. The strategies developed in this work could be used to improve the expression of other therapeutic proteins that are difficult to express. The superior enzymatic properies of the purified fibase suggest that it has potential applications for the therapy and/or prevention of thrombosis.

## Supplementary Information

**Additional file 1: Fig. S1.** Sequence of optimized and original *fib* gene. Upper row: the optimized *fib* sequence, lower row: the original *fib* sequence, different nucleic acids are marked with red.

**Additional file 2: Fig. S2.** Detection of *fib* copy number in the *K. phaffii* genome via a double standard curve method. (A) and (B) are the melting curves of *TDH1* and *fib* genes, and (C) and (D) are the amplification curves of *TDH1* and *fib* genes.

**Additional file 3: Fig. S3.** Lineweaver–Burk plots for purified fibrinolytic enzyme using chromogenix as a substrate. The experiment was repeated three times independently.

**Additional file 4: Table S1.** Important parameters during the entire fermentation process.

## Data Availability

All the data presented in the article are available from the corresponding author upon reasonable request.

## Abbreviations

Fibase: Fibrinolytic enzyme
