# Supplementary figures and images for: An effective combination of codon optimization, gene dosage, and process optimization for high-level production of fibrinolytic enzyme in Komagataella phaffii (Pichia pastoris)
Source: BMC Biotechnol. 2020 Dec 4;20:63. doi: 10.1186/s12896-020-00654-7 (PMC7716587; doi:10.1186/s12896-020-00654-7)

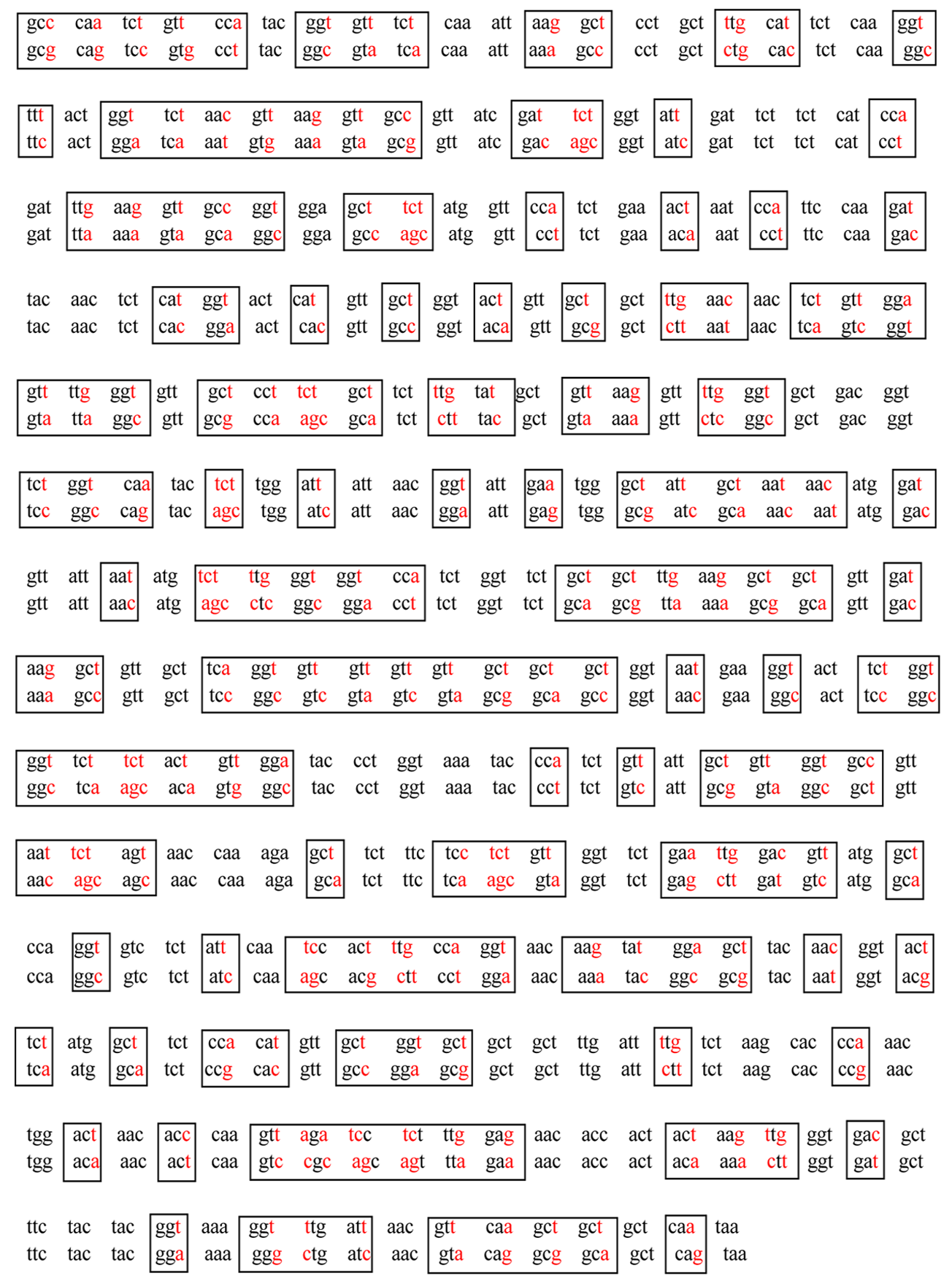

Supplement: Supplementary file 1 — Additional file 1: Fig. S1. Sequence of optimized and original fib gene. Upper row: the optimized fib sequence, lower row: the original fib sequence, different nucleic acids are marked with red. [file 12896_2020_654_MOESM1_ESM.tif]

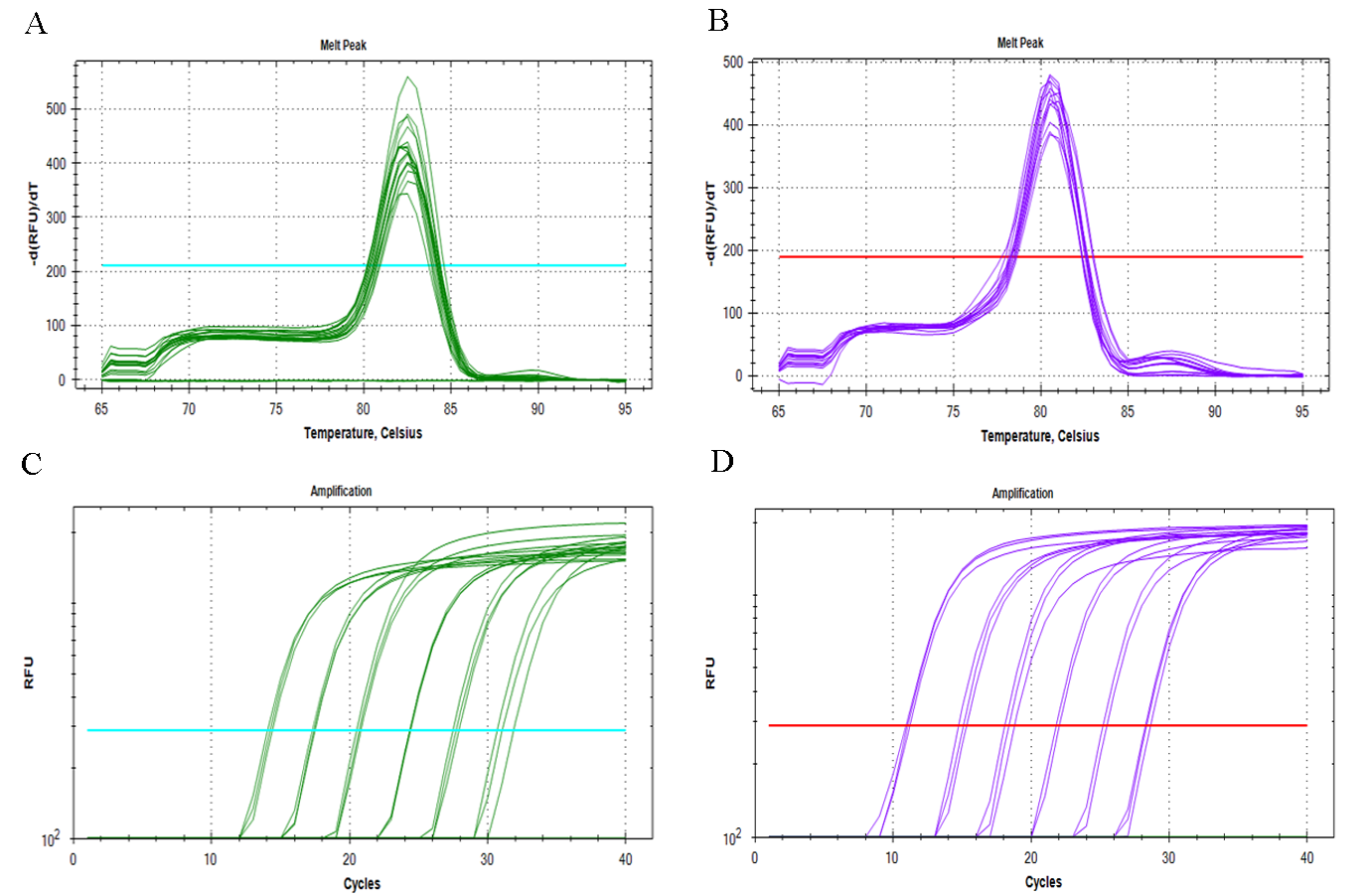

Supplement: Supplementary file 2 — Additional file 2: Fig. S2. Detection of fib copy number in the K. phaffii genome via a double standard curve method. (A) and (B) are the melting curves of TDH1 and fib genes, and (C) and (D) are the amplification curves of TDH1 and fib genes. [file 12896_2020_654_MOESM2_ESM.tif]
