# Supplementary material for: An effective combination of codon optimization, gene dosage, and process optimization for high-level production of fibrinolytic enzyme in Komagataella phaffii (Pichia pastoris)
Source: BMC Biotechnol. 2020 Dec 4;20:63. doi: 10.1186/s12896-020-00654-7 (PMC7716587; doi:10.1186/s12896-020-00654-7)
Supplement: Supplementary file 4 — Additional file 4: Table S1. Important parameters during the entire fermentation process. [file 12896_2020_654_MOESM4_ESM.docx]

**Table S1** Important parameters during the entire fermentation process.

| Fermentation parameters |  |
| --- | --- |
| Temperature | 32°C |
| inoculum size | 10% |
| Dissolved oxygen | > 20% |
| Original pH | 6.0 |
| Stirring speed | 240-450 r/min |
| Ventilatory volume | 2.5 L/min |
| Amount of defoamer added | 0.75‰ |
| Growth medium | glucose-free YPD |
| Induction medium | glucose-free YPD |
